# Patterns of nodal spread in stage III NSCLC: importance of EBUS-TBNA and ^18^F-FDG PET/CT for radiotherapy target volume definition

**DOI:** 10.1186/s13014-021-01904-4

**Published:** 2021-09-15

**Authors:** Maja Guberina, Kaid Darwiche, Hubertus Hautzel, Christoph Pöttgen, Nika Guberina, Thomas Gauler, Till Ploenes, Lale Umutlu, Dirk Theegarten, Clemens Aigner, Wilfried E. E. Eberhardt, Martin Metzenmacher, Marcel Wiesweg, Rüdiger Karpf-Wissel, Martin Schuler, Ken Herrmann, Martin Stuschke

**Affiliations:** 1 Department of Radiation Therapy, University Hospital Essen, West German Cancer Center, University Duisburg-Essen, Essen, Germany; 2 Department of Pulmonary Medicine, Section of Interventional Pneumology, University Medicine Essen – Ruhrlandklinik, West German Cancer Center, University Duisburg-Essen, Essen, Germany; 3 Department of Nuclear Medicine, University Hospital Essen, West German Cancer Center, University Duisburg-Essen, Essen, Germany; 4 German Cancer Consortium (DKTK), Partner Site University Hospital Essen, Essen, Germany; 5 Department of Thoracic Surgery and Thoracic Endoscopy, University Medicine Essen – Ruhrlandklinik, West German Cancer Center, University Hospital Essen, University Duisburg-Essen, Essen, Germany; 6 Institute of Diagnostic, Interventional Radiology and Neuroradiology, University Hospital Essen, University Duisburg-Essen, Essen, Germany; 7 Institute of Pathology, University Hospital Essen, West German Cancer Center, University Duisburg-Essen, Essen, Germany; 8 Department of Medical Oncology, University Hospital Essen, West German Cancer Center, University Duisburg-Essen, Essen, Germany; 9 Division of Thoracic Oncology, University Medicine Essen – Ruhrlandklinik, West German Cancer Center, University Duisburg-Essen, Essen, Germany

**Keywords:** NSCLC, Stage III, Lymphatic drainage, Pattern of spread, ^18^F-FDG PET/CT, EBUS-TBNA, Radiation

## Abstract

**Purpose:**

The aim of this study was to compare the pattern of intra-patient spread of lymph-node (LN)-metastases within the mediastinum as assessed by ^18^F-FDG PET/CT and systematic endobronchial ultrasound-guided transbronchial-needle aspiration (EBUS-TBNA) for precise target volume definition in stage III NSCLC.

**Methods:**

This is a single-center study based on our preceding investigation, including all consecutive patients with initial diagnosis of stage IIIA-C NSCLC, receiving concurrent radiochemotherapy (12/2011–06/2018). Inclusion criteria were curative treatment intent, ^18^F-FDG PET/CT and EBUS-TBNA prior to start of treatment. The lymphatic drainage was classified into echelon-1 (ipsilateral hilum), echelon-2 (ipsilateral LN-stations 4 and 7) and echelon-3 (rest of the mediastinum, contralateral hilum). The pattern of spread was classified according to all permutations of echelon-1, echelon-2, and echelon-3 EBUS-TBNA findings.

**Results:**

In total, 180 patients were enrolled. Various patterns of LN-spread could be identified. Skip lesions with an involved echelon distal from an uninvolved one were detected in less than 10% of patients by both EBUS-TBNA and PET. The pattern with largest asymmetry was detected in cases with EBUS-TBNA- or PET-positivity at all three echelons (*p* < 0.0001, exact symmetry test). In a multivariable logistic model for EBUS-positivity at echelon-3, prognostic factors were PET-positivity at echelon-3 (Hazard ratio (HR) = 12.1; 95%-CI: 3.2–46.5), EBUS-TBNA positivity at echelon-2 (HR = 6.7; 95%-CI: 1.31–31.2) and left-sided tumor location (HR = 4.0; 95%-CI: 1.24–13.2). There were significantly less combined ipsilateral upper (LN-stations 2 and 4) and lower (LN-station 7) mediastinal involvements (16.8% of patients) with EBUS-TBNA than with PET (38.9%, *p* < 0.0001, exact symmetry test). EBUS-TBNA detected a lobe specific heterogeneity between the odds ratios of LN-positivity in the upper versus lower mediastinum (*p* = 0.0021, Breslow-Day test), while PET did not (*p* = 0.19).

**Conclusion:**

Frequent patterns of LN-metastatic spread could be defined by EBUS-TBNA and PET and discrepancies in the pattern were seen between both methods. EBUS-TBNA showed more lobe and tumor laterality specific patterns of LN-metastases than PET and skipped lymph node stations were rare. These systematic relations offer the opportunity to further refine multi-parameter risk of LN-involvement models for target volume delineation based on pattern of spread by EBUS-TBNA and PET.

**Supplementary Information:**

The online version contains supplementary material available at 10.1186/s13014-021-01904-4.

## Introduction

Precise detection of the loco-regional pattern of tumor spread is of utmost importance for delineating the radiation target volume. The primary goal is to maximize effectiveness of radiotherapy while minimizing the treated volume in order to spare surrounding normal tissue. The balancing act between the optimal therapeutic benefit and possible long-term sequelae is a key issue in radiotherapy.

Evidence from older randomized trials with ^18^F-FDG PET-scans did not show a higher effectiveness of radiotherapy including elective nodal irradiation over involved field radiotherapy alone in locally advanced non-small cell lung cancer (NSCLC) [1, 2]. However, staging in these trials did not meet current standards and only a minority of patients received a pretreatment ^18^F-FDG PET/CT.

First the PET-plan trial was able to confirm the non-inferiority of involved field lymph node irradiation compared to a conventional target group including limited elective nodal irradiation at the primary endpoint of locoregional progression [3]. Hence, PET-based target volume delineation is a standard in radiotherapy planning. Here the gross tumor volume (GTV) includes PET-positive lymph nodes (LN) that will be expanded by 5–8 mm or up to an anatomic boundary to yield the clinical target volume (CTV) [4, 5].

Nevertheless, mediastinal LNs are known to be PET-positive also due to non-malignant causes. Acute or chronic infectious and inflammatory processes may result in LN enlargement with an elevated ^18^F-FDG-uptake, such as granulomatous inflammation, necrosis, as well as lymphoid infiltrates and anthracotic macrophages [6]. Due to the considerable high false discovery rate (FDR) of ^18^F-FDG PET/CT in comparison to histopathologic results, EBUS-TBNA with pathologic confirmation of mediastinal involvement is recommended in potentially curable NSCLC [7, 8].

In our preceding per lymph node analysis at this institution’s lung cancer database, we found a rising FDR of ^18^F-FDG PET/CT from echelon-1, to echelon-2 and -3 lymph nodes in patients with locally advanced NSCLC, treated with neoadjuvant or definitive radiochemotherapy [9]. There are some common patterns of lymphatic spread in NSCLC. Lobe specific patterns of spread of lymph node metastases in mediastinum are well-known from surgical series [10, 11]. Occult micrometastases may be regionally widely spread into the contralateral mediastinum, especially for left-sided tumors [12, 13]. Skip lesions from the primary tumor carried by lymphatic vessels directly into the mediastinum without involvement of the hilar LNs are less frequent [14, 15].

In the present study, we analyzed the agreement of the intra-patient pattern of nodal spread by EBUS-TBNA as well as PET, from the primary tumor to the upper and contralateral mediastinum. A prognostic model was built for echelon-3 involvement by EBUS depending on the pattern of spread revealed by PET and EBUS at the more proximal LN stations and the localization of the primary tumor. In addition, differences in the lobe-specific pattern of spread detected by EBUS and PET were assessed.

## Materials and methods

All consecutive patients with histopathologically proven NSCLC stage IIIA-C (according to AJCC/UICC/TNM 8th edition) presented in an academic lung cancer center for radiation oncology from December 2011 to June 2018 were enrolled in this study. This is a further evaluation of the exact intra-patient pattern of nodal spread complementing our previous study with a per lymph node analysis over all patients [9].

In all patients classified for a potentially curative concept at initial diagnosis, EBUS-TBNA and ^18^F-FDG PET/CT diagnostic information was obtained before start of treatment. Mandatory exclusion criterion was a previous cancer diagnosis or corresponding treatment.

After intravenous injection of 250–400 MBq ^18^F-FDG, PET/CT imaging was performed on the PET/CT Biograph mCT scanner (Siemens Healthineers, Germany). Two certified board members created the principal nuclear medicine report (PET-report): a nuclear medicine physician and a radiologist. SUV_max_ measurements were conducted in all EBUS-TBNA sampled LN stations, both for EBUS-positive (with tumor cells proven) and EBUS-negative (without a pathological proof of malignant cells).

EBUS-TBNA was regularly done in a systematic manner. All detectable LN stations larger than 5 mm (11-12L, 10-12R, 7, 4L, 4R, 2L, 2R) were sampled and entered into the study database according to the definition of the IASLC (International Association for the Study of Lung Cancer) lymph node map [16]. PET-positivity or negativity was also analysed per lymph node station. The LNs were grouped into echelons 1–3 as follows: (i) from the ipsilateral hilum as the first echelon (echelon-1), (ii) over the ipsilateral central mediastinum, i.e. LN stations 7 and ipsilateral LN station 4 as the second echelon (echelon-2) and (iii) to the upper ipsilateral mediastinum at LN station 2 or the contralateral mediastinal LN stations 2 and 4 and including the contralateral hilum as the third echelon (echelon-3) [9, 17, 18]. Therefore, echelon-3 comprises all EBUS-accessible lymph node stations that define N3 involvement in the mediastinum according to the 8^th^ TNM classification, as well as ipsilateral involvement of station 2. This classification also allows sub-analyses of the different stations of echelon-3 involvement depending on the involvement of echelon-1 and -2.

PET-positivity or EBUS-positivity was assigned to an echelon if at least one LN was positive in that echelon. At lymph node stations 5 and 6, surgical staging plays a decisive role for the exact staging before resection, especially in patients with EBUS-TBNA negative mediastinum, CT-morphologically indolent but PET-positive LN stations 5 and 6 or with primary tumor location in the left upper lobe [16, 19–21]. Because LN stations 5 and 6 are not routinely accessible by bronchoscopic intervention and transbronchial biopsy, they were not considered in this study for comparison of intra-patient spread patterns of LN metastases obtained by EBUS-TBNA and ^18^F-FDG PET/CT. Patterns of spread were classified according to echelon-1, echelon-2, and echelon-3 positivity or negativity by EBUS-TBNA or ^18^F-FDG PET/CT.

This study was approved by the local Ethics committee of the Medical Faculty (19-9056-BO).

Statistical analysis was performed using SAS software version 9.4, SAS/STAT 14.3 (SAS, Institute, Cary, NC) [22]. The procedures LOGISTIC, CORR, and FREQ were applied. All *p*-values are provided for two-sided hypotheses.

## Results

A total of 180 patients met the inclusion criteria of this study. Patients’ characteristics along with numbering/ranking of patterns of spread are shown in Table 1. The notational system for numbering of pattern of lymph node spread by PET/EBUS is based on the following sampling criteria: (i) patterns 1.1–1.6: discovery group 1 of patients with EBUS-TBNA samples from all three echelons-1 to -3 (104 patients), (ii) patterns 2.1–2.4: validation group 2 of patients with EBUS-TBNA samples for which EBUS samples are missing only at echelon-1 (37 patients) and (iii) patterns 3.1–3.5: validation group 3 of the remaining patients with EBUS-TBNA samples for which EBUS samples are missing at echelon-3 (39 patients).

**Table 1:** Patient characteristics

Patient characteristics	Number of patients
Histology	
Adeno-Ca	83
Squamous Cell Ca	80
Other	17
cT-category	
T1	18
T2	35
T3	53
T4	74
Pattern of lymph node spread by EBUS/PET: Discovery group 1 of patients with EBUS-TBNA samples from all echelons: n = 104	
1.1: echelon-1, 2, 3: negative, negative, negative (EBUS/PET)	4/4
1.2: echelon-1, 2, 3: positive, negative, negative (EBUS/PET)	31/21
1.3: echelon-1, 2, 3: positive, positive, negative (EBUS/PET)	52/37
1.4: echelon-1, 2, 3: negative, positive, negative (EBUS/PET)	3/2
1.5.1: echelon-1, 2, 3: negative, negative, positive (EBUS/PET)	0/1
1.5.2: echelon-1, 2, 3: positive, negative, positive (EBUS/PET)	1/4
1.5.3: echelon-1, 2, 3: negative, positive, positive (EBUS/PET)	0/1
1.6: echelon-1, 2, 3: positive, positive, positive (EBUS/PET)	13/34
Pattern of lymph node spread by EBUS/PET: Validation group 2 of patients with EBUS-TBNA samples for which EBUS samples are missing at echelon-1: n = 37	
2.1: echelon-2, 3: negative, negative (EBUS/PET)	15/10
2.2: echelon-2, 3: positive, negative (EBUS/PET)	17/15
2.3: echelon-2, 3: negative, positive (EBUS/PET)	1/2
2.4: echelon-2, 3: positive, positive (EBUS/PET)	4/10
Pattern of lymph node spread by PET/ EBUS: Validation group 3 of the remaining patients with EBUS-TBNA samples for which EBUS samples are missing at echelon-3: n = 39	
3.1: echelon-1, 2: negative/missing, negative (EBUS/PET)	3/2
3.2: echelon-1, 2: positive, negative (EBUS/PET)	12/9
3.3: echelon-1, 2: positive, positive (EBUS/PET)	14/19
3.4: echelon-1, 2: negative/missing, positive (EBUS/PET)	4/3
3.5: echelon-2: Patients with EBUS-untested echelon-2	6
RT-intent	
Definitive RT/CTx	114
Neoadjuvant RT/CTx	66
Laterality of the primary tumor	
Left-sided	83
Right-sided	93
Bilateral primaries	4
Tumor localization	
Upper or middle lobe alone	63
Lower lobe alone	27
Centrally or more than one lobe	90

Age	Median and range (years)
Median	62.9
Range	43.6–84.0

All numbers represent patients’ counts, except in the rows with patients’ age

**Fig. 1:**
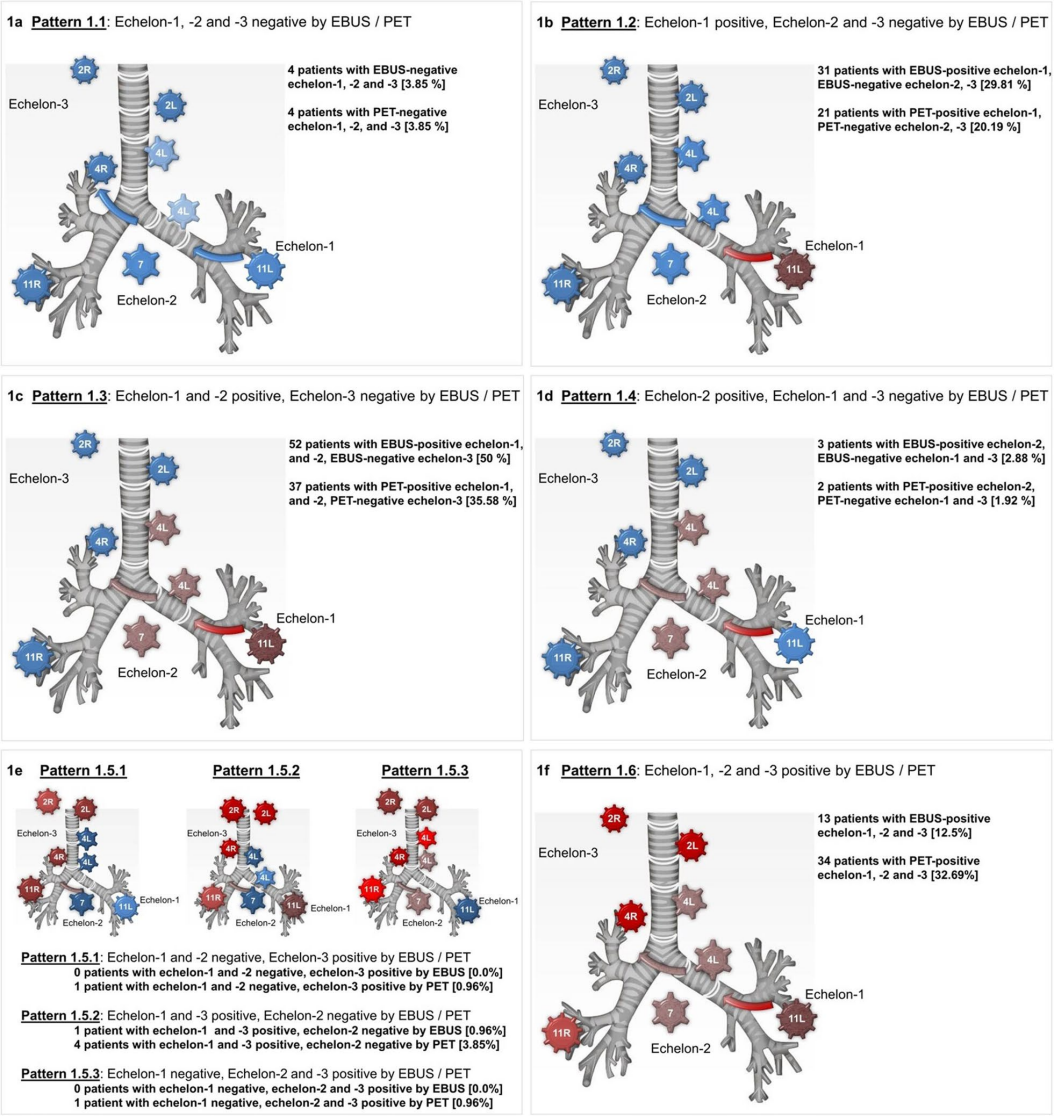
Graphical presentation of the various common patterns of nodal spread, distribution by means of EBUS-TBNA and PET/CT. Patterns of lymph node spread by EBUS/PET: Pattern 1.1: Echelon-1, -2, -3: negative, negative, negative (EBUS/PET); Pattern 1.2: Echelon-1, -2, -3: positive, negative, negative (EBUS/PET); Pattern 1.3: Echelon-1, -2, -3: positive, positive, negative (EBUS/PET); Pattern 1.4: Echelon-1, -2, -3: negative, positive, negative (EBUS/PET); Pattern 1.5.1: Echelon-1, -2, -3: negative, negative, positive (EBUS/PET); Pattern 1.5.2: Echelon-1, -2, -3: positive, negative, positive (EBUS/PET); Pattern 1.5.3: Echelon-1, -2, -3: negative, positive, positive (EBUS/PET); Pattern 1.6: Echelon-1, -2, -3: positive, positive, positive (EBUS/PET)

The aim of this study was to evaluate the concordance in the intra-patient pattern of spread in those LN echelons which can be assessed by both methods, EBUS-TBNA and ^18^F-FDG PET/CT. PET-positivity was observed in 72.2%, 67.8% and 32.8% of patients at echelon-1, -2, and -3, respectively. EBUS-TBNA samples were taken from echelon-1, -2, and -3 in 139, 174 and 144 patients, respectively. The prevalence of EBUS-positive echelons in PET-positive echelons that were analyzed by EBUS-TBNA was 99.2%, 81.8%, and 29.1% in echelon-1, -2, and -3, respectively. The intra-patient spread of LN metastases detected by EBUS-TBNA or PET across echelon-1 to -3 was classified into patterns 1.1–1.6, as shown in Table 1 and Fig. 1.

**Fig. 2:**
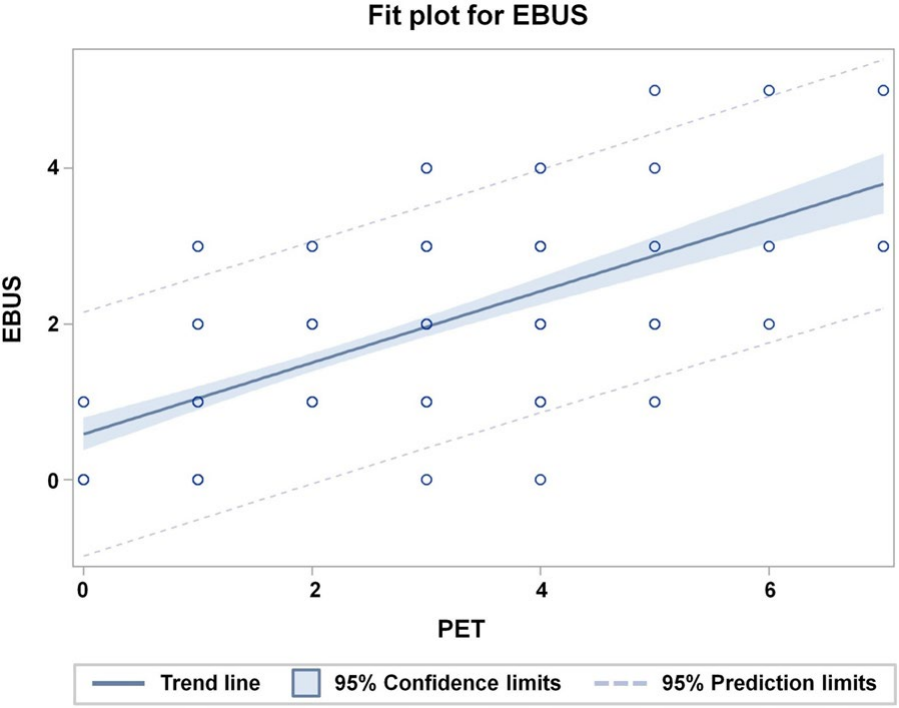
Fit plot for the dependence of the number of EBUS-positive lymph nodes per patient on the number of PET-positive lymph nodes per patient

The cross-tabulation of the involved LNs per patient according to EBUS- and PET-criteria excluding LN stations 5 and 6 per patient is depicted in Table 2. The correlation between the number of PET-positive and EBUS-positive LNs was distinct (Spearman correlation coefficient r_s_ = 0.67; 95%-CI: 0.56–0.77). The dependence of the number of EBUS-positive LNs on the number of PET-positive LNs per patient is shown in Fig. 2. The average slope of 0.46 ± 0.04, clearly less than 1 can be interpreted as an indication that several PET-positive LNs per patient could not be confirmed by EBUS (Fig. 2). The average slope for the 104 patients with EBUS-TBNA samples taken from all 3 echelons was 0.44 ± 0.05.

**Table 2:** Cross-tabulation of EBUS and PET positive nodes in EBUS-accessed lymph node stations per patient in the overall group of 180 patients

Number of PET-positive LNs	Number of EBUS-positive LNs					
	0	1	2	3	4	5
0	14	2	0	0	0	0
1	7	32	3	2	0	0
2	0	16	35	2	0	0
3	1	5	18	7	2	0
4	1	4	6	6	2	0
5	0	2	4	1	1	1
6	0	0	1	1	0	1
7	0	0	0	2	0	1

All numbers represent patient counts. Spearman correlation coefficient r_s_ = 0.63 (95% CI: 0.53–0.71)

**Fig. 3:**
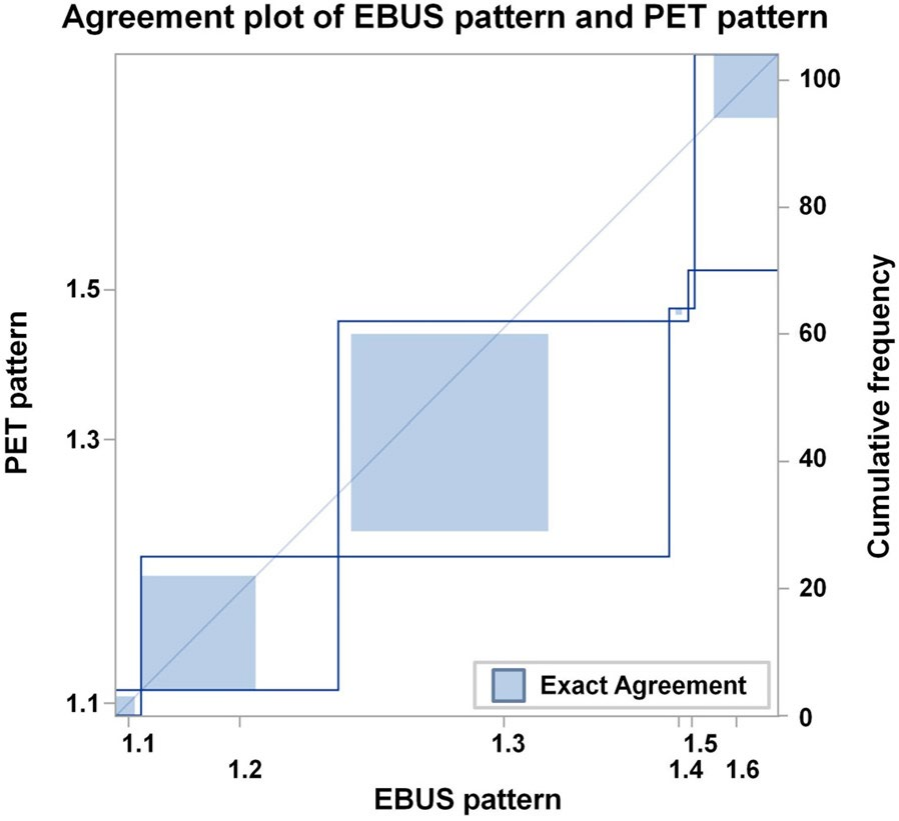
Agreement plot of EBUS-TBNA samples and PET/CT results from all three echelons 1–3. Note: Agreement-plot of pattern of spread of lymph node metastases according to EBUS-TBNA and PET. Pattern of spread are ordered according to the classification given in Table 1. The cumulative frequencies according to the EBUS-pattern and PET-pattern are plotted. The step heights represent frequencies of the respective pattern according to EBUS and PET. The blue dashed squares represent the frequencies of patients with agreement of the indicated pattern.

Table 3 shows the cross-tabulation of the patterns by EBUS-TBNA and PET for the 104 patients with EBUS samples from all three echelons. Figure 3 shows on the abscissa the cumulative frequencies of patients with pattern of involvement up to the indicated pattern class according to EBUS-TBNA and on the ordinate to PET. This agreement plot shows significant deviations from symmetry (*p* < 0.0001, exact symmetry test). The weighted kappa coefficient as a measure of inter-staging agreement between two examination methods is κ = 0.37 (95%-CI: 0.23–0.51).

**Table 3:** Cross-tabulation of pattern of lymph node spread according to EBUS-TBNA (EBUS-pattern) and PET (PET-pattern) in the 104 with EBUS samples from all three echelons

EBUS	PET						
	PET Pattern 1.1	PET Pattern 1.2	PET Pattern 1.3	PET Pattern 1.4	PET Pattern 1.5	PET Pattern 1.6	Sum over rows
EBUS Pattern 1.1	3	0	0	0	1	0	4
EBUS Pattern 1.2	0	18	4	1	3	5	31
EBUS Pattern 1.3	0	2	31	0	1	18	52
EBUS Pattern 1.4	1	0	0	1	1	0	3
EBUS Pattern 1.5	0	0	0	0	0	1	1
EBUS Pattern 1.6	0	1	2	0	0	10	13
Sum over columns	4	21	37	2	6	34	104

All numbers represent patient counts

Subsequently, we analyzed which of the patterns showed significant differences in symmetry and found significant asymmetry in patterns 1.2 (*p* = 0.013, exact symmetry test), 1.3 (*p* = 0.0039, exact symmetry test) and 1.6 (*p* < 0.0001, exact symmetry test). These results are shown in Fig. 3. Skip lesions, i.e. patterns 1.4 and 1.5 with an involved echelon distal from an uninvolved echelon, were seen in less than 10% of patients by both EBUS-TBNA and PET on a per patient analysis. There were no significant differences in the frequency of skip lesions between the two methods (*p* = 0.056, exact symmetry test).

Table 3 shows that 53% of PET pattern 1.6 patients were classified as pattern 1.3 by EBUS-TBNA, so that an echelon-3 involvement following an echelon-1 and echelon-2 involvement could not be confirmed by EBUS-TBNA in the majority of patients. This observation is associated with the asymmetry seen for pattern 1.3. With respect to the asymmetry for pattern 1.2, 29% of EBUS pattern 1.2 patients were upgraded to patterns 1.3 and 1.6 by PET, because a PET positive echelon-2 LN could not be confirmed by EBUS.

As a validation cohort, we analyzed the pattern of spread in the 37 patients with EBUS-sampled echelon-2 and -3 but with an untested echelon-1 (Table 1). When analyzing the asymmetry in the pattern of spread again, we found more patients with PET-positivity at echelon-2 and -3 than with EBUS-TBNA-positivity at both echelons (*p* = 0.014, exact symmetry test). In the remaining 39 patients, no asymmetries were observed between the examination methods across patterns 3.1–3.4.

Table 1 indicates that for the 141 patients with EBUS-TBNA samples in echelon-2 and -3, patterns of positive echelon-3 lymph nodes were significantly more frequently observed with PET than with EBUS-TBNA, i.e. in 52 from 141 vs. 19 from 141 patients (*p* < 0.0001, *χ*^2^-test). Therefore, we analyzed the dependence of EBUS-positivity in echelon-3 on factors determining the intra-patient pattern of lymphatic spread by PET and EBUS in echelons more proximal to the primary tumor, i.e. PET-positivity in echelon-1, -2 and -3 as well as EBUS-positivity in echelon-1 and -2, and laterality of the primary tumor as prognostic factors using univariable and multivariable logistic regression with forward selection. EBUS-positivity at echelon-2, PET-positivity in echelon-3 and laterality became significant prognostic factors in univariable and multivariable analysis for EBUS-positivity in echelon-3. The respective odds ratios (OR) for EBUS positivity in echelon-3 are shown in Table 4. The fraction of patients with EBUS-positivity at echelon-3 having an EBUS-positive echelon-2 was 19.1% (95%-CI: 11.5–28.8%) versus 3.9% (95%-CI: 0.5–13.2%) with an EBUS-negative echelon-2. The fraction of echelon-3 EBUS-positives among echelon-3 PET-positives was 58.8% (95%-CI: 35.4–82.2%) for the 17 patients with left-sided tumors and echelon-2 EBUS-positivity, 11.1% (95%-CI: 0.0–31.2%) for the 9 patients with left-sided tumors and echelon-2 EBUS-negativity and 19.2% (95%-CI: 4.1–4.4%) for the 27 patients with right-sided tumors. The dependence of echelon-3 EBUS-positivity on echelon-2 EBUS-positivity indicates a continuous instead a skipping lymphatic spread. 

A supplementary analysis revealed that 97% of the echelon-3 lymph nodes examined were located in the contralateral mediastinum or hilum and only 3% were ipsilateral station 2 lymph nodes, with the former defined as N3 involvement and the latter as N2 involvement according to the 8^th^ TNM classification. All factors that were found to be significant for echelon-3 involvement by EBUS-TBNA or PET using multivariable logistic regression were also found to be predictive of N3 involvement when repeated with the same logistic regression model. The respective odds ratios (OR) for N3 involvement using multivariable analysis are shown in Additional file 1: Table 4S.

**Table 4:** EBUS-positivity in echelon-3: Respective odds ratios (OR) according to prognostic factors from univariable and multivariable analysis

Significant prognostic factors	EBUS positivity in echelon 3			
	Univariable analysis		Multivariable analysis	
	OR (95%-CI)	*p*-value*, χ*^2^-test	OR (95%-CI)	*p*-value*, χ*^2^-test
PET-positivity vs. negativity in echelon-3	12.7 (3.5–46.4)	0.0001	12.1 (3.2–46.5)	0.0003
EBUS-positivity vs. negativity in echelon-2	5.9 (1.3–26.7)	0.021	6.7 (1.31–31.2)	0.022
Laterality (left-sided tumors compared with right-sided)	2.8 (1.01–7.9)	0.049	4.0 (1.24–13.2)	0.020

The respective odds ratios for EBUS-positivity in echelon-3 according to the prognostic factors from univariable and multivariable logistic regression analysis were: (i) OR = 12.7 (95%-CI: 3.5–46.4) (*p* = 0.0001, *χ*^2^-test) and OR = 12.1 (95%-CI: 3.2–46.5) (*p* = 0.0003, *χ*^2^-test) in dependence on PET-positivity vs. negativity in echelon-3, (ii) OR = 5.9 (95 CI: 1.3–26.7) (*p* = 0.021, *χ*^2^-test) and OR = 6.7 (95%-CI: 1.31–31.2) (*p* = 0.022, *χ*^2^-test) in dependence on EBUS-positivity vs. negativity in echelon-2, as well as (iii) OR = 2.8 (95%-CI: 1.01–7.9) (*p* = 0.049, *χ*^2^-test) and OR = 4.0 (95%-CI: 1.24–13.2) (*p* = 0.020, *χ*^2^-test) for left-sided tumors compared with right-sided

In addition, we analyzed the dependence of the probability of PET-positivity in echelon-3 on PET-positivity in echelon-1 and -2 and laterality of the primary tumor as prognostic factors and found that PET-positivity in echelon-2 alone was significant by multivariable logistic regression with forward selection (odds ratio for PET-positivity in echelon-3 as a function of PET-positivity vs. PET-negativity in echelon-2 is shown in Additional file 1: Table 4S: OR = 4.2 (95%-CI: 1.7–10.3), *p* = 0.0012, *χ*^2^-test).

Of the 75 patients with right-sided tumors and EBUS-proven echelon-2 and -3, 6 had echelon-3 EBUS-positivity, 3 of them at LN station 2R, and 3 at station 4L. Twenty-six of these 75 patients had PET-positivity at echelon-3, 13 at station 2R, 13 at station 4L, 6 in the contralateral hilum and 2 at station 2L. Of the PET-positives at stations 2R, 4L and the contralateral hilum, 3, 2 and 0 were EBUS-positive at their respective stations.

Of the 66 patients with left-sided tumors and EBUS-proven echelon-2 and -3 LNs, 13 had echelon-3 EBUS-positivity, all of them at LN station 4R, 1 also at station 10R, and 1 also at station 2R. Twenty-six of these 66 patients had PET-positivity at echelon-3, 20 at station 4R, 1 at station 2R and 9 in the contralateral hilum. Of the PET-positives at stations 4R, 2R, and the contralateral hilum, 11, 0 and 1 were EBUS-positive at these stations, respectively. The respective fraction of EBUS-positives among PET-positives at station 4R for left-sided tumors was 11/20 (55%) and for the remaining left-sided and the right-sided PET-positive patients at echelon-3, EBUS-positivity at echelon-3 was observed on average in 5/32, i.e.15.6% (*p* = 0.003, Cochrane-Mantel–Haenszel test).

In addition, we analyzed the dependence of the pattern of lymphatic spread by EBUS and PET on the lobe of the primary tumor and therefore used a grouping of LN zones underlying lobe-specific LN dissection, i.e. in the lower mediastinum (station 7), the ipsilateral upper mediastinum (ipsilateral stations 4 and 2) and the contralateral mediastinum/ hilum [11]. With regard to the involvement of the upper or lower ipsilateral mediastinum, there were marked asymmetries by EBUS and PET (*p* < 0.0001, exact symmetry test). In particular, there was significantly less combined upper and lower mediastinal involvement (16.8%) with EBUS compared to PET (38.9%), (*p* < 0.0001, exact symmetry test). EBUS detected more involvement at the lower mediastinum in lower-lobe tumors (53.3%) than in upper/middle lobe tumors (25.0%), (*p* = 0.044, Cochrane-Mantel–Haenszel test). In parallel, EBUS detected more upper mediastinal involvement in upper/middle lobe tumors (61.4%) than in lower lobe tumors (26.7%), (*p* = 0.021, Cochrane-Mantel–Haenszel test). The odds ratio of EBUS-positivity in the upper mediastinum compared to the lower mediastinum differed significantly between upper and middle lobe tumors (OR = 4.8; 95%-CI: 1.9–11.9) and lower lobe tumors (OR = 0.51; 95%-CI: 0.34–0.78) (Breslow-Day test for homogeneity of odds ratios: *p* = 0.0021). This could not be defined for PET (Breslow-Day test for homogeneity of odds ratios: *p* = 0.19). The contralateral mediastinum at stations 4 and 2 was significantly more frequently involved by EBUS-TBNA for left-sided tumors than for right-sided tumors (20% vs. 4%, *p* = 0.0035, Cochrane-Mantel–Haenszel test). This difference was not observed by PET (*p* = 0.11, Cochrane-Mantel–Haenszel test).

Additional discrepancies between invasive staging and PET/CT may exist in stations 5 and 6, but no data are available from this series. In the present study, 37 patients had PET-positive lymph nodes at stations 5 and 6. Only 2 of them had right-sided tumors and echelon-2 involvement was detected in both by EBUS-TBNA. Nineteen patients had PET-positive stations 5 and 6 as well as a negative mediastinal EBUS-TBNA at echelon-2 and -3. Seventeen of these patients had EBUS-positive ipsilateral hilar lymph node metastases and an enlargement of the target volume to stations 5 and 6 was considered moderate. These data show that the potential risk of a false-positive PET-finding at stations 5 and 6 to change the radiation target volume by adding more volume is rather low.

## Discussion

This is a large consecutive case-series study at an academic lung cancer center demonstrating the potential change of radiotherapy protocols depending on primary diagnostic procedures EBUS-TBNA and ^18^F-FDG PET/CT for NSCLC staging. LN staging pathways are based on the knowledge of lymphatic drainage and mediastinal anatomy.

Research work on lymphatic drainage originates from surgical, pathological and anatomical studies and, more recently, from imaging techniques. The mediastinal N2 status is the subject of a broad investigation in lung cancer. It represents a major challenge in the clinic and requires profound clinical diagnostics of mediastinal spread followed by consequent selection of the most appropriate local therapy [23, 24]. The International Association for the Study of Lung Cancer (IASLC) has recently proposed a more detailed breakdown of the LN staging system [25]. Prognostic factors depending on LN staging are a central topic of lung cancer research [26, 27]. Several studies examined the potential relevance depending on the position and extent of the LN infestation [17, 18, 28]. Riquet et al. showed on anatomical examinations that the lymph node drainage presents a lymphatic chain and functional entity which channels the lymph into the systemic circulation. The authors stated that the term chain should be used rather than LN station, because the chain contains the prognostic meaning as a whole. These studies referred to the work of I. Caplan, who defined 3 mediastinal regions (in relation to the tracheoesophageal axis) for both the upper and the lower mediastinum [28].

Here we followed the classification based on study results of Riquet et al. and I. Caplan, which we also used in our previous work [9]. The clinical target volume was defined upon both diagnostic procedures EBUS-TBNA and ^18^F-FDG PET/CT. When considered independently, neither modality provided exact information alone about the spread of LN metastases. Combining these two modalities allowed defining the spread of LN metastasis more precisely.

Primary, this study detected significant differences in the intra-patient pattern of lymph node involvement defined by EBUS-TBNA and PET/CT. A pattern with all lymph node echelons involved was found significantly more often by PET than by EBUS-TBNA.

In addition, EBUS-TBNA revealed a higher frequency of contralateral mediastinal involvement for left-sided tumors compared to right-sided tumors and a preferential involvement of ipsilateral superior mediastinal lymph nodes of upper and middle lobe tumors compared to lower lobe tumors with a preferential involvement of subcarinal nodes. These patterns were not detected by PET diagnostics. Skip lesions were seen in less than 10% of patients in this study with both staging procedures, however at a high prevalence of hilar lymph node metastases with both methods.

Such lobe and side specific spread of lymph node metastases as found in this study by EBUS-TBNA were also detected in specimens from large surgical series [10, 11, 29]. Nohl-Oser pointed out a markedly higher risk of contralateral mediastinal involvement in left-sided compared to right-sided carcinomas [10]. Watanabe et al. described lobe-specific pattern of spread into the ipsilateral mediastinum and developed a strategy of selective nodal dissection based on these data [11]. However, with more advanced multi-station LN involvement, combined ipsilateral superior and inferior involvement is common, so that the lobe specificity is lost [30]. In addition, the recurrence pattern after surgery with and without postoperative radiotherapy shows a specific pattern of spread depending on the primary tumor location [31]. Micrometastases in mediastinal lymph nodes were more frequently observed than metastases by conventional histopathology and also in early stages with a negative impact on prognosis [7, 12, 32, 33].

In this study, a model of the risk of LN metastatic involvement of the echelon-3 lymph nodes in the EBUS-TBNA was built as a function of PET-positivity in proximal lymph node stations, as well as EBUS positivity in proximal lymph node stations and laterality of the primary tumor location. Factors that were dependent on the pattern of spread became significant in this model. Sophisticated models for determining the probability of malignant involvement, based on multi-parameter imaging and minimally invasive spread diagnostics, have the potential to improve the accuracy of target volume delineation in radiotherapy planning of stage III NSCLC. This allows a further refinement of the current standard of including all PET-positive lymph nodes in the target volume [4, 5]. PET/CT positivity carries the risk of false positive results [6–9]. The FDR of PET/CT generally increases with decreasing prevalence of truly involved lymph nodes and was found to be approximately 45% at a prevalence of 20% and approximately 34% at a prevalence of 25% of N2/N3 lymph nodes in per patient analysis [34, 35]. According to the results of the presented study, this risk depends on the respective echelon-1 to -3 of the considered lymph node station, the localization of the primary tumor and the pattern of spread observed by EBUS-TBNA. The risk level of lymph node involvement accepted for inclusion of a lymph node station in the target volume also depends on the local control probability at the primary tumor achieved with contemporary radiochemotherapy schedules as well as the increase of the risk of normal tissue toxicity by inclusion of an additional lymph node station. The closer the plan is to the accepted tolerance limits, the less likely a lymph node station will be included in the target volume in order not to exceed these limits. Isolated in-field recurrences are found in current studies in about 20–50% of patients after 5 years [36, 37].

The negative predictive value of EBUS-TBNA in PET-positive lymph nodes is of particular importance for the exclusion of a lymph node station from the target volume. The negative predictive value was 91% for PET-positive nodes in the study of Bauwens et al. (2008) at a prevalence of mediastinal lymph node metastases of 58% and 89% in the study of Taverner et al. (2016) at a prevalence of 11% [38, 39]. However, the negative predictive value of EBUS-TBNA was considerably lower in the study of Rintoul 2009 at 63% on a per patient basis [40], but given the well-known relationship between negative predictive value and prevalence [41], this might be dependent on the very high prevalence of lymph node metastases of 88% in that study. According to this relation and assuming a sensitivity of EBUS-TBNA of ≥ 80% and a specificity of 100%, the negative predictive value of EBUS-TBNA approaches values above 93% at a prevalence of involved LNs of less than 25% in PET-positive lymph nodes. From these studies, a negative EBUS-TBNA predicts a low risk of lymph node involvement in PET-positive lymph nodes at a low to moderate underlying prevalence of true involvement. A group from Manchester constructed a logistic model to predict nodal false negativity of EBUS-TBNA. Standardized uptake value of lymph nodes, standardized uptake ratio between lymph node and primary tumor, and heterogeneous echogenicity were found as significant risk factors [42].

There are some limitations in this study. PET-positivity at stations 5 and 6 was not included in further analysis as these LN stations were not accessible with EBUS-TBNA. To correctly detect mediastinal involvement in the left para-aortic mediastinum, a preoperative minimal surgical staging may be a necessary diagnostic step [8, 19].

Our investigation points out systematic asymmetries between the intra-patient patterns of lymph node spread in stage III NSCLC detected by EBUS-TBNA and PET. For a single lymph node station, an assessment of the risk of involvement depending on the pattern of lymphatic metastases detected by PET and by EBUS-TBNA in other lymph node echelons can help to include or exclude these regions in the target volume. As genuine for a radiotherapy study, we did not have surgical assessment to validate the spread pattern by EBUS-TBNA. Surgical staging is only available for selected cases. An additional mediastinoscopy cannot generally reduce the false negative rate by EBUS-TBNA, provided that EBUS-TBNA is performed by an experienced team [43].

In summary, morphologically inconspicuous PET-positive echelon-3 lymph nodes should still be assessed critically. If the prevalence in a given lymph node station is known to be low with respect to the localization of the primary tumor and the pattern of LN spread by PET/CT and EBUS-TBNA, then the negative predictive value of EBUS-TBNA is so high that it should be considered for radiotherapy planning. Furthermore, this study points out the low risk of involvement of PET-positive echelon-3 lymph node stations depending on the pattern of more proximal nodal spread.

With the knowledge gained from the combined EBUS-TBNA and ^18^F-FDG PET/CT information, it is possible to identify the intra-patient spread of lymph node metastases along the lymphatic chain in mediastinum. PET/CT does not only function as a search test and EBUS-TBNA as a confirmatory test. Moreover, both diagnostic tools complement each other, and combined may further adjust the target volume.

## Conclusion

This study underlines the importance of combined PET/CT and EBUS-TBNA diagnostics for radiotherapy treatment planning. Mediastinal patterns of lymph node metastases differed by EBUS-TBNA and PET. EBUS-TBNA showed a lobe and tumor laterality specific pattern of spread and skipping of echelon-2 was rarely seen. These systematic relations provide the opportunity to further refine multi-parameter risk models for lymph node involvement to delineate target volume based on patterns of spread by EBUS-TBNA and PET.

## Supplementary Information

**Additional file 1: Table 4S.** The respective odds ratios (OR) for EBUS-positivity in echelon-3 excluding ipsilateral LN station 2 according to the prognostic factors from multivariable analysis

## Data Availability

All data generated and analyzed during this study are included in this published article (supplementary information can be received from the corresponding author.

## Abbreviations

^18^F-FDG: 2-Deoxy-2-[^18^F]fluoro-d-glucose
PET: Positron-emission-tomography
CT: Computed-tomography
NSCLC: Non-small cell lung cancer
CTV: Clinical target volume
LN: Lymph nodes
GTV: Gross tumor volume
FDR: False discovery rate
EBUS-TBNA: Endobronchial ultrasound-guided transbronchial-needle aspiration
HR: Hazard ratio
OR: Odds ratio
CI: Confidence interval
RT: Radiotherapy
CTx: Chemotherapy
