# Supplementary material for: Patterns of nodal spread in stage III NSCLC: importance of EBUS-TBNA and 18F-FDG PET/CT for radiotherapy target volume definition
Source: Radiat Oncol. 2021 Sep 15;16:176. doi: 10.1186/s13014-021-01904-4 (PMC8442338; doi:10.1186/s13014-021-01904-4)
Supplement: Supplementary file 1 — Additional file 1: Table 4S. The respective odds ratios (OR) for EBUS-positivity in echelon-3 excluding ipsilateral LN station 2 according to the prognostic factors from multivariable analysis [file 13014_2021_1904_MOESM1_ESM.docx]

| **Table 4S** | The respective odds ratios (OR) for EBUS-positivity in echelon-3 excluding ipsilateral LN station 2 according to the prognostic factors from multivariable analysis |
| --- | --- |

| **EBUS positivity in echelon 3 excluding ipsilateral LN station 2**  **Significant prognostic factors** | **Multivariable analysis** | |
| --- | --- | --- |
|  | **OR (95%-CI)** | ***p*-value*, χ*^2^-test** |
| PET-positivity vs. negativity in echelon-3 | 13.0 (3.1–53.9) | 0.0004 |
| EBUS-positivity vs. negativity in echelon-2 | 14.8 (1.7–131) | 0.016 |
| Laterality (left-sided tumors compared with right-sided) | 8.2 (1.9–35.6) | 0.005 |
| PET-positivity vs. negativity in echelon-2 | 4.2 (1.7–10.3) | 0.0012 |

Note:

The respective odds ratios for N3 involvement using multivariable analysis were: (i) OR = 13.0 (95% CI: 3.1–53.9) (*p* = 0.0004, *χ*^2^-test) in dependence on PET-positivity vs. negativity in echelon-3, (ii) OR = 14.8 (95 CI: 1.7–131) (*p* = 0.016, *χ*^2^-test) in dependence on EBUS-positivity vs. negativity in echelon-2, (iii) OR = 8.2 (95 CI: 1.9–35.6) (*p* = 0.005, *χ*^2^-test) for left-sided tumors compared with right-sided and (iv) OR = 4.2 (95 -CI: 1.7–10.3) (*p* = 0.0012, *χ*^2^-test) in dependence on PET-positivity vs. negativity in echelon-2.
